# Traumatic Humeral Shaft Non-union With Ulnar Nerve Transection: An Orthobiologics Success Story

**DOI:** 10.7759/cureus.35189

**Published:** 2023-02-19

**Authors:** Christopher Williams, Travis Redmond, Cleo Stafford, Walter Sussman

**Affiliations:** 1 Physical Medicine and Rehabilitation, Interventional Orthopedics and Regenerative Medicine, Interventional Orthopedics of Atlanta, Atlanta, USA; 2 Physical Medicine and Rehabilitation, Emory University School of Medicine, Atlanta, USA; 3 Orthopedics, Emory University School of Medicine, Atlanta, USA; 4 Physical Medicine and Rehabilitation, Tufts Medical Center, Atlanta, USA; 5 Sports Medicine, Boston Sports and Biologics, Atlanta, USA

**Keywords:** ulnar nerve transection, humeral fracture, bmac, orthobiologics, platelet lysate, platelet rich plasma, bone marrow aspirate concentrate, non-union

## Abstract

Long bone non-union is a detrimental, yet common condition that affects many individuals after injury. It can lead to significant pain and weakness that may impact lifetime productivity and quality of life. This report describes a patient who suffered from greater than two years of a distal humerus fracture non-union along with an ulnar nerve transection that failed traditional surgical management and underwent a percutaneous injection with bone marrow aspirate concentrate, platelet-rich plasma, and platelet lysate, demonstrating subsequent fracture resolution and strength improvement.

## Introduction

Humeral shaft fractures are relatively common, representing approximately 1% to 5% of all fractures and 20% of all humeral fractures [1,2]. Non-operative management of humeral shaft fractures has been described with good results, with some studies reporting greater than 90% union rates and acceptable functional outcomes [1]. Other reports suggest non-union rates as high as 27.5% with non-operative care [3], and a greater association with malunion. The rate of non-union is approximately 2% but can be as high as 20% for diaphyseal fractures depending on the injury type. A fracture can be classified as a non-union when there has been a failure of union observed over nine months or failure to progress over the preceding three months [4].

The following case report details a patient who suffered an open humeral shaft fracture and ulnar nerve transection. His recovery was complicated by non-union and was managed surgically; however, the patient did not have a resolution of his fracture or functional deficiencies. In this case, after multiple failed surgical attempts, the patient underwent a bone marrow aspirate concentrate (BMAC) injection at the site of non-union, and platelet-rich plasma with platelet lysate injection and hydrodissection of the ulnar nerve.

## Case presentation

**Figure 1:**
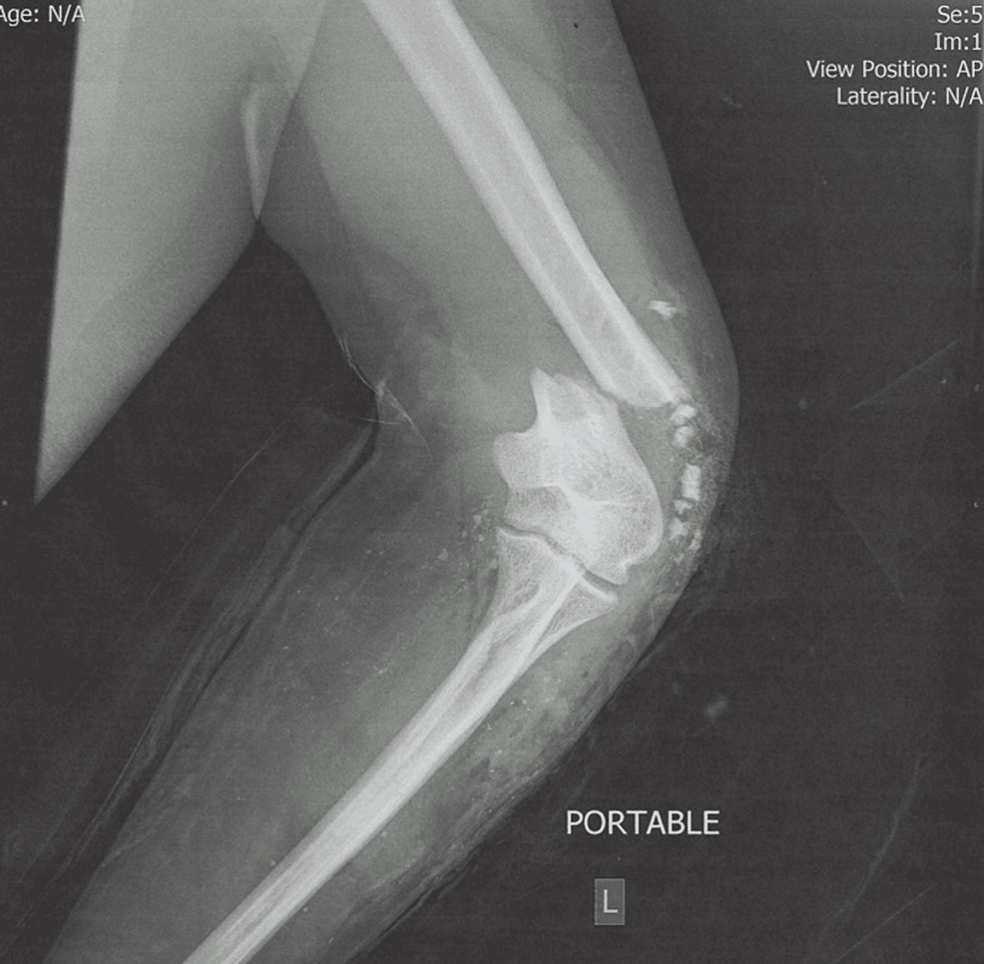
Initial X-ray after trauma demonstrating comminuted fracture of the distal humerus

A 16-year-old male with no past medical history was involved in a rollover motor vehicle accident in which the vehicle flipped onto the driver’s side, pinning the patient’s left arm between the door and the road. The patient was taken to a local trauma center where he was found to have a left open distal humerus transverse fracture, left upper extremity degloving injury (wound >50 cm^2^), and a left ulnar nerve transection (Figure 1). He underwent an emergent wound washout and open reduction and fixation of the humerus with crossed pins followed by internal fixation one week later. The postoperative course was complicated by a wound infection requiring incision and drainage (I&D) with hardware removal one week following the internal fixation. He had two subsequent I&Ds.

**Figure 2:**
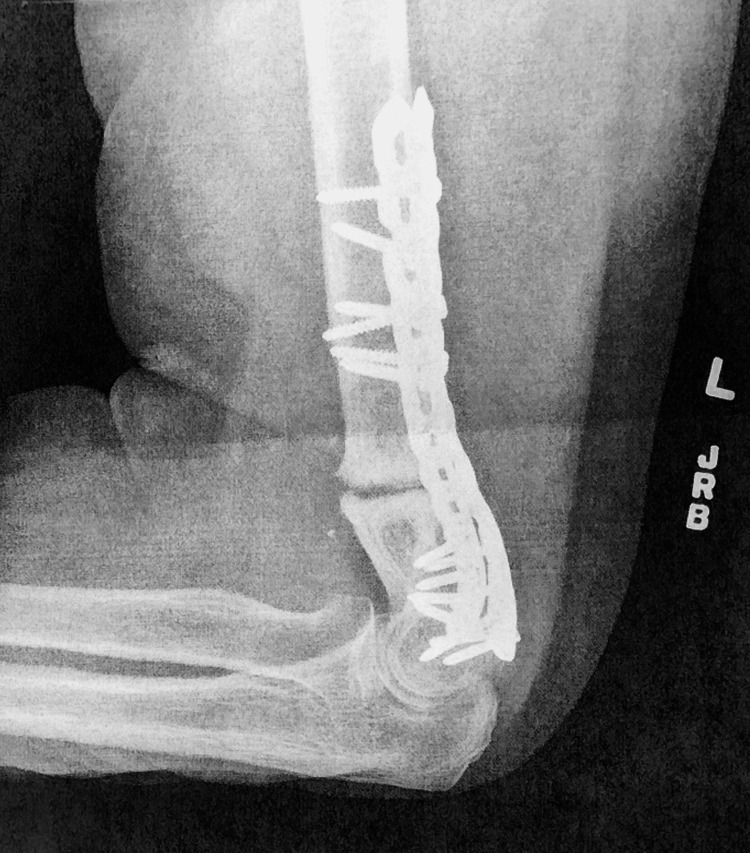
Follow-up X-ray 25 months after injury displaying chronic non-union and three months prior to the orthobiologic procedure

Approximately one month after the accident, the patient underwent an ulnar nerve reconstruction using a nerve allograft (4.5 mm diameter x 135 mm length) and a split-thickness skin graft of the posterior arm wound (11x3.5 cm) and forearm wound (15x8 cm). Two months after the accident, the patient underwent an irrigation, debridement, and antibiotic spacer placement with tobramycin cement for the bone fragments. Approximately five months after the injury another I&D, removal of hardware, and a revision ORIF was performed with the placement of additional antibiotic drug delivery. Six months after the accident, an I&D and non-union repair with femoral bone allograft harvest was performed. Despite these efforts, there was persistent non-union of the humeral shaft fracture at follow-up and a second bone allograft was implanted 12 months later by the surgical team. At the 25-month follow-up visit, there was still persistent non-union despite the second bone allograft (Figure 2).

The patient presented to our clinic approximately two years following the accident with persistent non-union of the humeral shaft fracture and symptoms of pain in the arm and weakness of the hand muscles. The patient had a full range of motion at the elbow but was unable to lift objects greater than five pounds due to pain. X-rays showed a distal humerus fracture with hypertrophic callus formation but no significant osseous bridging. A nerve conduction study (NCS) was notable for prolonged ulnar motor distal onset latency (7.9 ms), reduced amplitude (0.1 mV), and decreased conduction velocity with drop off across the elbow (Below Elbow-Wrist 37 m/s and Above Elbow-Below Elbow 25 m/s).

There was prolonged peak latency of the dorsal ulnar cutaneous sensory nerve action potential (SNAP) (3.0 ms) and the ulnar SNAP was absent. Electromyography of the first dorsal Interosseous muscle was significant for increased insertional activity, moderately increased spontaneous activity, and diminished recruitment. Findings were consistent with acute and chronic ulnar denervation proximal to the elbow.

**Figure 3:**
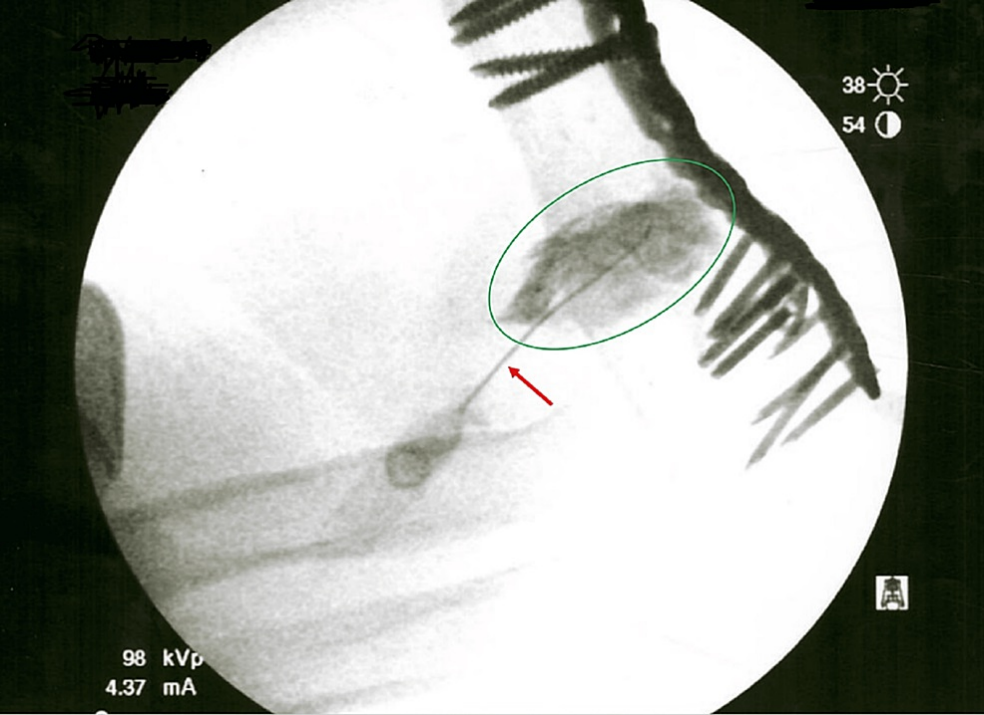
Fluoroscopic-guided image with the utilization of a 22 gauge 3.5 inch needle for the placement of contrast material and the biologic injectate into the humeral non-union site Yellow circle = contrast/injectate. Red arrow = needle.

Treatment options were discussed with the patient, including percutaneous biologic injections utilizing bone marrow aspirate concentrate (BMAC) injection, and the patient elected to proceed with a biologic injection combing the use of BMAC, platelet-rich plasma (PRP), and platelet lysate (PL) into the site of non-union. In preparation for the procedure, the patient was instructed to avoid any non-steroidal anti-inflammatory drugs for at least 10 days prior and corticosteroids for six weeks prior to the procedure date. On the day of the procedure, the bone marrow was aspirated and the platelet products were prepared by techniques that have been previously described [5,6]. In brief, bone marrow was harvested from the bilateral iliac crest under sterile conditions utilizing ultrasound guidance. Following local anesthesia with 1% lidocaine and 0.25% ropivacaine, an 11-gauge bone marrow biopsy needle was utilized to obtain 40 cc of bone marrow aspirate (BMA) from three locations along the posterior superior iliac spine (PSIS) on each side of the pelvis, for a total of 80 cc. The BMA was hand-processed in a sterile ISO-7 cleanroom under ISO-5 laminar flow cabinets. The buffy coat was first isolated through centrifugation, producing 1-5 mL of BMC. This was transported sterilely to the procedure room for use. Approximately 60 cc of venous blood was drawn into anticoagulated yellow top tubes for the preparation of the PRP and PL. The PRP was prepared with 200x g centrifugation to separate the plasma and buffy coat from the red blood cells (RBC). The subsequent remaining supernatant was RBC- and leukocyte-poor. The PL was prepared in a similar fashion of centrifugation and the supernatant was manually extracted in a sterile hood via pipette and placed in a -20⁰ freezer for approximately 30 minutes or until frozen and then thawed followed by re-centrifugation of the thawed pellet and platelet bodies. The supernatant is then extracted under the sterile hood for utilization with a pipette. The BMAC was processed to a final volume of 6 cc with a total nucleated cell count of 1.99 billion and viability of 89%, as determined by a Bio-Rad TC-20 automated cell counter (Bio-Rad Laboratories, Inc., Hercules, CA). Under fluoroscopic guidance, 4 cc of BMAC, 1 cc of PRP, and 1 cc of PL mixture were injected into the site of the non-union between the fracture fragments under fluoroscopic guidance using a 22-g 3.5-in needle (Figure 3). The patient also underwent an ulnar nerve hydrodissection at the cubital tunnel utilizing a 27-g 1.5-inch needle with a 6 cc injectate mixture of processed PRP/PL under ultrasound guidance.

**Figure 4:**
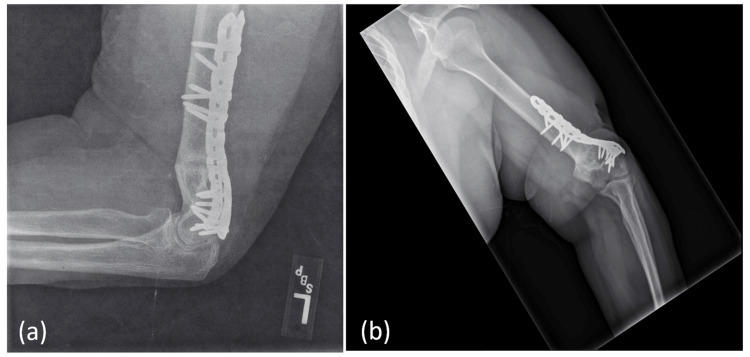
(a) X-ray image three months and (b) image 11 months post-procedure demonstrating complete fracture healing

Following the procedure, the patient was encouraged to start a progressive rehabilitation program with occupational therapy and avoid non-steroidal anti-inflammatory drugs (NSAIDs) or corticosteroids for a minimum of six weeks post-treatment. The patient demonstrated progressive gains in function as measured by grip strength (initial evaluation 24 lb; final 30 lb), key pinch (initial evaluation 0.5 lb; final: 12 lb), three-point grip (initial evaluation 0.5 lb, final 5 lb), and tip pinch (initial evaluation 1 lb; final 7.5 lb). A post-procedural X-ray was obtained approximately four months after the procedure and showed a healed distal humerus fracture (Figure 4) and an electromyography (EMG)/nerve conduction study (NCS) performed approximately six months after the procedure demonstrated a minimal change from the prior study.

## Discussion

Non-union of long bone fractures can occur in 2-20% of cases [7]. The Food and Drug Administration (FDA) defines non-union as nine months of an unhealed fracture and at least three consecutive months with no progression of fracture healing. In cases of failed surgical management options are limited. In this case, the patient failed multiple surgical attempts at treating the humeral shaft non-union but responded positively to a biologic injection that consisted of a combination of BMAC, PRP, and PL into the site of non-union under image guidance. There were no complications observed during the follow-up period post-procedure. Limitations include this being a single case report and some additional considerations are listed below.

Fracture healing

Long bone non-union can present a significant problem, and the ‘diamond concept’ has been presented as a conceptual framework for what is essential for successful bone healing. Risks of non-union include patient-dependent and independent factors. Ku et al. (2022) identified several risk factors for non-union of distal humerus fractures after open reduction and internal fixation (ORIF) [8]. Elevated body mass index (BMI), diabetes mellitus status, combined fractures, and tension band wiring fixation were all associated with an increased risk of non-union in distal humeral fractures. Although our patient did not exhibit any of the aforementioned risk factors, Nicholson et al. describe several others that are relevant to our patient, such as high energy mechanism of injury, open fracture, fully displaced fracture, and infection [4], all of which may have contributed to his humeral non-union.

Successful fracture healing is dependent on the biological environment at the fracture site and the optimum mechanical environment. Healing is primarily a biological process that remains dependent upon the cellular response and the cellular microenvironment. The ‘diamond concept’ gives equal importance to mechanical stability and the biological environment, and management decisions should take into account the availability of osteoinductive mediators, osteogenic cells, an osteoconductive matrix (scaffold), optimum mechanical environment, adequate vascularity, and addressing any existing comorbidities of the host.

In this case, a percutaneous biologic injection consisting of BMAC, PRP, and PL was used as a direct means of concentrating mesenchymal stromal cells (MSCs) to act as osteoprogenitors and as a source of other osteoinductive substances derived from the PRP and PL [9]. BMAC has been successfully used to augment surgical fixation to treat long bone non-union fractures with the ‘diamond concept’ or BMAC alone [10,11], but to the author's knowledge, BMAC has not been used to augment non-operative management. BMAC can serve as a reliable source of MSCs [12], which are thought to differentiate into osteogenic cells [13]. This case redemonstrates the efficacy of BMAC to promote fracture healing in a complicated case of non-union after failed surgical fixation and grafting of an upper extremity fracture.

Neuroregeneration

Platelet-rich plasma and platelet lysate are thought to create a favorable environment for neuroregeneration by serving as a fibrin scaffold and releasing neurotrophic/neurotropic growth factors/molecular signals (i.e. brain-derived neurotrophic factor, insulin-like growth factor 1, platelet-derived growth factor, vascular endothelial growth factor, hepatocyte growth factor, fibrin, fibronectin, and vitronectin) [14]. In vitro, platelet-rich plasma has also been shown to promote the elongation of Schwann cells [15].

PRP-aided nerve regeneration has been demonstrated in vivo by Garcia de Cortazar et al. (2018), after a young male suffered a radial nerve transection and underwent a series of intraneural PRP injections four months post-injury and achieved functional recovery [14]. Additionally, multiple randomized trials and several case reports have demonstrated efficacy in the treatment of median nerve neuropathy at the wrist (i.e. carpal tunnel syndrome) utilizing PRP with observed improvements in pain/functional measures, nerve swelling, and electrophysiologic findings as well [16]. Although the neural recovery of our patient has not been as robust given the length of the nerve defect (13.5 cm), delay in surgical repair (>1 month), and duration of time prior to treatment with platelet products (>2 years), he has had significant functional improvement in terms of strength, pain reduction, and quality of life. Further studies should be performed in ideal circumstances to assess the therapeutic potential of PRP and PL in post-traumatic neuroregeneration.

## Conclusions

Bone marrow aspirate concentrate has shown to be highly successful in cases of long bone non-union. This case report highlights the efficacy of BMAC with platelet products in the setting of a complex injury with a chronic non-union and ulnar nerve transection. Platelet-rich plasma and platelet lysate may be beneficial to promote neuroregeneration after a peripheral nerve injury; however, the severity and chronicity of nerve injury should be considered. Further studies, including randomized clinical trials, are warranted to investigate the clinical utility and broader applicability for the treatment of complex cases of non-union.
